# Linear interictal pain in Epicrania Fugax: a reply

**DOI:** 10.1186/s10194-015-0506-8

**Published:** 2015-03-17

**Authors:** Yu Wang, Qing-Qing Pan, Ya-Nan Lu, Miao-Miao Tian, Xian-Hong Wang

**Affiliations:** Department of Neurology, Epilepsy and Headache Group, the First Hospital of Anhui Medical University, Jixi Road 218, 230022 Hefei, China

**Keywords:** Linear headache, Epicranial fugax, Interictal pain, Primary headache

## Abstract

This is a reply to the comments on our article “Linear headache: a recurrent unilateral head pain circumscribed in a line-shaped area” published in JHP 2014 Jun 26; 15:45. In the comments, the authors raise a question whether the linear headache (LH) we reported be a linear interictal pain in epicranial fugax (EF), based on a case they reported. We think that the LH is not a linear interictal pain in EF based on our observations and considerations.

## Correspondence

We thank Dr. Juan A. Pareja and Dr. Pablo Bandrés for their comments on our article [1]. They raise a question whether the linear headache (LH) we reported be a linear interictal pain in epicranial fugax (EF) while this interictal pain become the main complaint, based on a interesting case they encountered [2].

We read with great interest the case description by Pareja and Bandrés and, in a general impression, agree that the continous pain in between EF is an interictal pain associated with EF in this case as the localization of the continous pain was the same as that of EF and this pain was milder than the EF pain itself in severity. These two features are also the critical features of the interictal pain of other primary headache syndromes [3]. Yes, this interictal pain and the EF pain described by Pareja and Bandrés topographically parallels to that in some of our LH patients. From the descriptions of our reported patients [1] and unreported patients, the LH pain areas are mainly localized in a lineal area linking the occipitocervical point and the internal *canthus* of the ipsilateral eye, with some in a lineal area little shorter than this “typical” linear trajectory in length. The LH pain area is similar to the pain trajectory of EF [4,5] and this was why we needed to differentiate it from EF in our article [1]. But, we do not think that the LH pain in our patients is an interictal pain of EF based on the following observations and considerations: 1) If the LH pain is an interictal pain of EF, the LH pain should, at least in some of our reported and unreported patients, be milder than the EF pain and the patients would describe the precedent ultrashort duration of more severe pain but the LH patients did not. And it is unreasonable to think that all the LH pain predominates the precedent EF pain and become the main complaint. In fact, we have made inquiry whether they ever had an EF pain for most patients and the response were all negative, though this initial inquiry was not aimed at exploring an antecedent pain in LH; 2) Though we have no available data concerning the medication response of the interictal pain in primary headaches including EF, it is hard to imagine that the LH pain had well response to medications used for migraine prophylaxis which implys a non-peripheral mechanism if the LH pain is an interictal pain in EF whose medication response implys a peripheral origin [5,6]; 3) The LH pain can occassionally cause ophthalmoplegia in its years of recurrence [7]. On the other hand, we might consider the possibility that the interictal pain in EF of Pareja and Bandrés is LH pain triggered by EF as EF can trigger the attacks of migraine and cluster headaches as we reported previously [8], but we need to know the detailed pain character description and medication response of this interictal pain in EF.

Thus, the clarification of the possible relationship between EF and LH will likely be approached by publication of additional clinical reports on the LH pain associated with EF as well as by more detailed mechanism investigations on LH and EF.

## Abbreviations

LH: Linear headache
EF: Epicranial fugax
